# Next-generation leukemia diagnostics: Integrating LC-MS/MS proteomics with liquid biopsy platforms

**DOI:** 10.1016/j.jlb.2025.100324

**Published:** 2025-08-08

**Authors:** Vivek Singh

**Affiliations:** Department of Biochemistry, King George's Medical University, Lucknow, Uttar Pradesh, 226003, India

**Keywords:** Liquid biopsy, Leukemia diagnosis, T-ALL, LC-MS/MS, Proteomics, Biomarker discovery, Circulating protein biomarkers, Multi-omics

## Abstract

Diagnosing leukemia often depends on invasive bone marrow biopsies, which can be painful and may fail to detect the early stages of the disease. Liquid biopsy, a minimally invasive method that analyzes circulating biomarkers in blood, has emerged as a powerful tool for the early detection of leukemia. Among emerging technologies, liquid chromatography-tandem mass spectrometry (LC-MS/MS) enables high-throughput and sensitive profiling of blood-based proteins, thereby creating new opportunities for biomarker discovery. This mini-review highlights the clinical potential of LC-MS/MS in liquid biopsy for leukemia, with a focus on T-cell acute lymphoblastic leukemia (T-ALL). Recent proteomic studies have identified distinct protein signatures in the blood of T-ALL patients, such as XRRA1, CPNE4, and S100A8, which show substantial diagnostic value. We also address similar applications in acute myeloid leukemia (AML), the challenges of clinical translation, and the future integration of proteomics with multi-omics diagnostic platforms. Importantly, we discuss the limitations of current studies (e.g., small cohorts, limited diversity, and reproducibility issues) and the path toward clinical implementation, including validation in larger trials, regulatory considerations, cost-effectiveness, and the need for standardized protocols. LC-MS/MS-driven liquid biopsy represents a promising advancement toward earlier, less invasive, and more precise leukemia diagnostics, provided that robust validation and harmonization efforts are successful.

## 1 Introduction

Leukemia is a heterogeneous group of hematological malignancies characterized by the clonal proliferation of immature blood cells. T-cell acute lymphoblastic leukemia (T-ALL), which arises from T-cell progenitors, accounts for approximately one-quarter of pediatric leukemia cases [1]. The conventional diagnosis of T-ALL and other leukemias relies on invasive bone marrow biopsies and extensive laboratory workups, including blood counts, immunophenotyping (flow cytometry), cytogenetic analysis (karyotyping and fluorescence in situ hybridization), and molecular testing for known gene rearrangements or mutations [2]. Although these methods are effective, they often necessitate invasive sampling and may only detect the disease after a significant tumor burden is present. Early-stage detection of leukemia remains challenging, yet it is crucial for improving patient outcomes. There is a pressing need for minimally invasive diagnostic approaches to identify leukemia-associated changes in the circulation before the onset of overt clinical disease.

Liquid biopsy has emerged as a promising approach to meet this need. Instead of relying on bone marrow aspirates or tissue biopsies, liquid biopsy detects cancer-derived biomarkers in easily accessible body fluids, primarily blood [3]. In solid tumors, liquid biopsies typically analyze circulating tumor DNA (ctDNA), circulating tumor cells (CTCs), or exosomal nucleic acids [4]. In leukemias, which inherently involve blood and marrow, the liquid biopsy concept extends to detecting leukemia-specific molecular signatures, including DNA, RNA, or proteins, in the peripheral blood [5]. By capturing these biomarkers, liquid biopsies could enable earlier diagnosis, real-time disease monitoring, and better prognostication with significantly less patient discomfort.

## 2 Liquid biopsy in leukemia

**Fig. 1:**
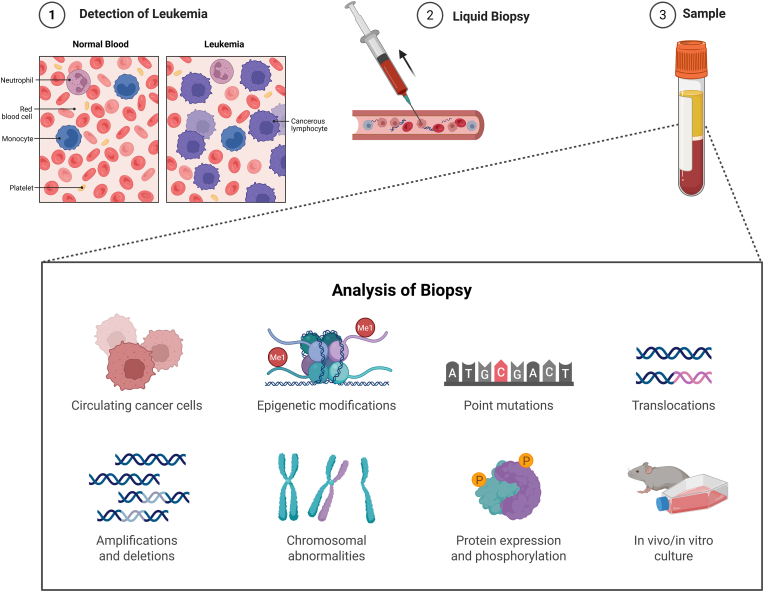
**Liquid Biopsy Workflow and Multi-Omics Analysis in Leukemia** Schematic representation of the liquid biopsy workflow for leukemia detection. (1) Comparison between normal blood and leukemic blood showing the presence of abnormal cancerous lymphocytes. (2) Blood draw as a minimally invasive method to obtain a sample for analysis. (3) Collection of peripheral blood, followed by multi-layered sample analysis, including circulating cancer cells, epigenetic modifications, point mutations, chromosomal abnormalities, gene amplifications/deletions, protein expression/phosphorylation, and in vivo/in vitro culture systems. These analyses offer a comprehensive molecular overview of leukemia, enabling early diagnosis and personalized monitoring.

Liquid biopsy offers multiple advantages in the context of leukemia management. It is minimally invasive, highly informative, and can be performed repeatedly over time [3]. This makes it ideal for monitoring disease dynamics and treatment response, potentially complementing or replacing traditional tissue biopsies [4]. Traditional bone marrow biopsies are not only painful and resource-intensive, but they also provide information from a single site that may not capture the full molecular heterogeneity of leukemia. In contrast, blood-based biomarkers can reflect systemic disease and allow serial sampling, which is valuable for tracking minimal residual disease (MRD) or early signs of relapse [4]. A variety of analytes can serve as biomarkers for liquid biopsy in leukemia. These include cell-free DNA fragments carrying leukemia-specific mutations or gene fusions, circulating leukemia cells or blast-derived exosomes, circulating microRNAs, and protein biomarkers. Notably, many leukemias have well-known genetic markers (for example, BCR-ABL1 in chronic myeloid leukemia or TEL-AML1 in pediatric ALL) that can be detected in blood plasma by sensitive PCR or sequencing techniques [5]. However, beyond genetic markers, the proteomic profile of blood is an emerging frontier for leukemia diagnosis. The circulating proteome (including proteins and peptides shed by leukemia cells or altered in the patient's blood due to malignancy) holds a wealth of diagnostic information [6]. Recent studies emphasize that analyzing circulating peptides and proteins in serum or plasma can significantly aid in diagnosing and stratifying acute leukemias, as shown in Fig. 1 [7]. Despite the relative scarcity of such proteomic studies in leukemias to date, the few reports available show promising results that can contribute to precision medicine in leukemia care [8].

## 3 LC-MS/MS technology

**Fig. 2:**
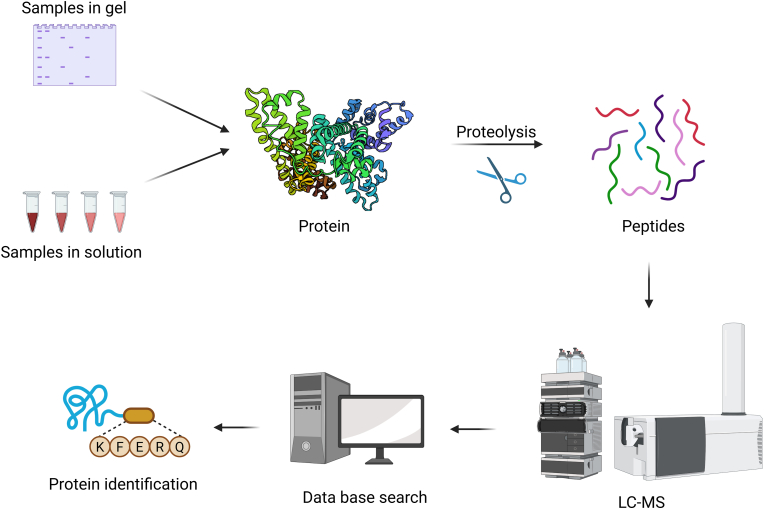
**Proteomic Workflow Using LC-MS/MS for Biomarker Discovery** Overview of the LC-MS/MS-based proteomic workflow used for leukemia biomarker discovery. Protein samples are obtained from gel or solution and subjected to proteolysis to generate peptides. These peptides are then analyzed by liquid chromatography-tandem mass spectrometry (LC-MS/MS). The resulting spectra are matched to protein databases for identification and quantification, enabling high-throughput, unbiased discovery of differentially expressed proteins in patient samples.

Liquid chromatography-tandem mass spectrometry (LC-MS/MS) is a powerful analytical technology at the core of modern proteomics. In the context of liquid biopsy, LC-MS/MS enables researchers to profile the protein composition of blood (or other fluids) in an unbiased and high-throughput manner, as illustrated in Fig. 2 [9,10]. The typical workflow for proteomic analysis involves digesting proteins into peptides, separating them by liquid chromatography, and then identifying and quantifying them using tandem mass spectrometry. Advanced mass spectrometers can detect thousands of peptides in a single run, enabling the identification of hundreds to thousands of proteins from a complex sample, such as plasma. This approach has been successfully applied to discover biomarkers for early detection, prognosis, and therapy monitoring in various diseases, including cancers [11]. One significant advantage of LC-MS/MS-based proteomics is its ability to provide unbiased multiplexing capability. Unlike antibody-based assays that target one protein at a time, LC-MS/MS can simultaneously survey a broad swath of the proteome without prior knowledge of which proteins might be necessary. This makes it ideal for biomarker discovery – uncovering novel protein markers that more limited assays would miss.

Furthermore, modern LC-MS/MS offers outstanding analytical specificity and sensitivity, often surpassing conventional immunoassays in the detection of low-abundance molecules [12]. Technology has also seen improvements in speed and throughput, resulting in reduced turnaround times for analyses, as shown in Table 1 [13]. In leukemia liquid biopsy, LC-MS/MS is used in a label-free or labeled quantitation mode to compare protein levels between patient samples and controls. Label-free quantitation (as employed in several recent studies) uses the MS signal intensity or spectral counts to infer protein abundance differences. In contrast, labeling techniques (such as TMT or iTRAQ) introduce tags for multiplexing samples in one run. Combined with robust bioinformatics, both approaches can pinpoint proteins differentially expressed in leukemia versus healthy states. The result is a panel of candidate biomarkers that can then be further investigated [[14], [15], [16], [17], [18], [19]].

**Table 1:** Summarizes some key advantages of LC-MS/MS for clinical proteomic applications.

S.No.	Types	Applications
1.	**Multiplex Detection**	Profile thousands of proteins/peptides in one sample run, providing a global view of the disease state.
2.	**High Specificity & Sensitivity**	Reliably distinguish protein isoforms and modifications with low detection limits, often surpassing traditional antibody-based assays in accuracy.
3.	**Minimal Sample Requirements**	Requires only small volumes of blood plasma/serum, making it suitable for repeat sampling in vulnerable patients.
4.	**Fast Turnaround**	Advances in instrumentation and automation have enabled rapid proteomic workflows, speeding up data acquisition and analysis.
5.	**Unbiased Discovery**	Does not require predefined targets, allowing discovery of unexpected biomarkers and pathways involved in leukemia.

## 4 Diagnostic applications of LC-MS/MS in leukemia

Researchers have begun to identify protein biomarkers in blood that could facilitate the early diagnosis of leukemia by using LC-MS/MS-based proteomics. This approach is particularly appealing for detecting aggressive subtypes, such as T-ALL, where early intervention is crucial. However, it is essential to note that proteomic investigations in leukemia are still in their early stages of development. A 2024 review highlighted that peptidomic studies in acute leukemias are relatively few but show great promise [20], underscoring the opportunity for discovery.

## 5 T-ALL early diagnosis

Proteomic Study by Singh et al. (2024): One of the landmark recent studies in this area is the work by Singh and colleagues, who applied label-free LC-MS/MS to identify novel protein markers for early-stage T-ALL. In their 2024 study, blood samples from 20 T-ALL patients (collected at the time of initial diagnosis, before chemotherapy) and 20 healthy controls were analyzed by LC-MS/MS to catalog and quantify proteins. The proteomic workflow identified 1467 proteins in the blood, among which 44 proteins were significantly dysregulated in T-ALL (nine >2-fold upregulated and 35 >2-fold downregulated in patients vs. controls). Notably, several disease-related proteins showed marked elevation in T-ALL, with some exceeding threefold higher abundance in patients compared to healthy individualsfor example, eleven-nineteen lysine-rich leukemia protein (ELL), triggering receptor expressed on myeloid cells 1 (TREM1), cisplatin resistance-associated overexpressed protein (CISOP), X-ray radiation resistance-associated protein 1 (XRRA1), tumor necrosis factor receptor superfamily member 10D (TNFRSF10D), protein S100-A8 (S100A8), and copine-4 (CPNE4). Many of these proteins are generally not detectable at high levels in healthy blood, forming a leukemia-specific protein signature [21]. Singh et al. went beyond discovery and performed initial validation of these findings. They used reverse transcription PCR (RT-PCR) to confirm that the genes encoding several top candidate proteins were upregulated at the mRNA level in T-ALL cells and ELISA to quantitatively measure the corresponding protein levels in an independent set of serum samples. The validation focused on several candidates, including XRRA1, TREM1, CPNE4, ELL, CISOP, TNFRSF10D, and S100A8. Consistently, these markers showed significant differences between T-ALL and control samples in the validation experiments. For instance, ELISA confirmed that proteins such as ELL, S100A8, TNFRSF10D, and CPNE4 were elevated approximately 3-fold in patient serum compared to controls (p < 0.0001). Moreover, the authors performed receiver operating characteristic (ROC) analysis, demonstrating that multiple candidates achieved excellent discriminative power (area under the ROC curve, AUROC (a statistical measure of diagnostic test accuracy, where 1.0 indicates perfect discrimination) approaching 0.97–1.0 for the top markers). Such high AUROC values indicate these proteins could reliably distinguish T-ALL patients from healthy individuals in this cohort [21]. The study concluded that these differentially expressed proteins constitute a T-ALL-specific “protein signature” and represent promising early diagnostic and prognostic biomarkers. It also provided a valuable proteomic map of T-ALL, laying the groundwork for further investigations. However, as the authors caution, these results are preliminary and require validation in larger patient cohorts.

**Limitations:** Indeed, the small sample size (20 patients) and single-center nature of the Singh et al. study present essential limitations. Early-stage discoveries, such as this, can overestimate biomarker performance due to cohort-specific idiosyncrasies or overfitting. For example, the remarkably high AUROC values achieved in a limited dataset may not hold up in broader populations or prospective studies. Therefore, these candidate protein markers must be validated in larger, independent cohorts that include more diverse patient populations and appropriate controls to ensure their robustness and clinical relevance. The initial follow-up by Singh et al., using PCR and ELISA, provided proof of concept; however, actual clinical utility will require multi-center trials and consistent results across different laboratories. In summary, while this T-ALL proteomic study is very promising, its findings should be viewed as hypothesis-generating. The results illustrate the potential of LC-MS/MS in leukemia diagnostics, but further validation work (including expanding the cohort size and confirming the markers in new patient sets) is necessary before any of these protein biomarkers could be considered for routine clinical use [17,18].

## 6 Proteomic biomarkers in AML

Similar LC-MS/MS-driven efforts have been conducted in acute myeloid leukemia (AML), another aggressive leukemia where new biomarkers are needed. Jajula et al. (2024) performed an integrative proteomic analysis comparing bone marrow interstitial fluid and serum from AML patients with those from healthy controls [22]. Using a label-free LC-MS/MS approach, they identified 201 proteins with altered abundance in the bone marrow fluid of AML patients and 123 dysregulated proteins in patient serum [22]. Bioinformatic pathway analysis of these proteins highlighted leukemia-related perturbations (e.g., changes in FAK signaling, IL-12 signaling, and macrophage production pathways) in AML samples [22]. Jajula et al. carried the discovery forward into the verification and validation phases. They employed a targeted multiple reaction monitoring (MRM; a targeted mass spectrometry technique selectively detecting and quantifying specific proteins with high sensitivity) MS assay to verify selected candidates in a separate cohort. They then validated a small biomarker panel via ELISA in an independent set of patient samples [22]. This process yielded a proposed three-protein panel consisting of pro-platelet basic protein (PPBP, also known as CXCL7), enolase 1 (ENO1), and beta-2-glycoprotein 1 (APOH), which were significantly elevated in AML patients’ samples compared to controls [22]. Notably, each of these proteins has a plausible link to leukemia biology (for example, CXCL7/PPBP is a platelet-derived factor that can modulate hematopoiesis, and ENO1 is a metabolic enzyme often upregulated in cancer cells). The three-protein panel could prove helpful in distinguishing AML cases and was suggested to aid in AML diagnosis and prognosis in the future [23].

**Limitations:** Similar to the T-ALL study, the findings from Jajula et al. are based on relatively limited patient cohorts and must be interpreted with caution. The discovery phase and subsequent MRM verification involved a specific set of patients, and the final ELISA validation was performed on a modest number of samples. While the multi-step approach (discovery → targeted verification → ELISA validation) is a strength, the number of patients in each phase was small. Thus, it remains to be proven whether the proposed PPBP–ENO1–APOH panel will generalize to the broader and highly heterogeneous AML patient population. AML comprises diverse subtypes with varying genetic and clinical characteristics; a three-protein signature might not capture all variants of the disease. Larger-scale validation studies are necessary to determine if this protein panel consistently differentiates AML from non-leukemic conditions across multiple cohorts and clinical settings. Such studies should include patients from diverse demographics and leukemia subtypes and ideally be conducted across various centers to assess inter-laboratory reproducibility.

Additionally, one must consider how this proteomic panel would complement existing diagnostics – for instance, would it add discriminatory power on top of current genetic tests or risk scores? Until these questions are addressed, the AML proteomic biomarkers reported by Jajula et al. should be viewed as promising leads that require further confirmation. Encouragingly, the authors demonstrated an ability to verify their markers using targeted MRM and ELISA assays [19], a critical step toward clinical translation. The next step will be to expand validation to larger cohorts and independent sample sets to build a case that these biomarkers have broad and reproducible utility in AML diagnosis.

## 7 Challenges and future directions

Translating LC-MS/MS proteomic findings into routine clinical leukemia diagnostics poses several significant challenges. These include the need for extensive biomarker validation, ensuring reproducibility across different laboratories, meeting regulatory requirements, demonstrating clinical utility in trials, and addressing practical issues such as cost and integration into healthcare. Below, we discuss these issues and potential solutions in detail.

## 8 Validation and reproducibility challenges

A primary hurdle in proteomic biomarker development is validation of candidates identified in discovery studies [17]. Discovery-phase proteomics often yields tens or even hundreds of molecules that appear differentially expressed between leukemia and control samples [21]. Narrowing this list to a reliable diagnostic panel requires extensive follow-up. It is usually not feasible to carry every candidate forward, so researchers must prioritize markers that are biologically plausible and technically feasible to measure in clinical labs (for example, proteins that are secreted into blood, or those for which high-quality antibodies or targeted MS assays exist) [22,23]. Even after prioritization, each candidate biomarker needs orthogonal verification typically using methods like targeted MS (e.g., MRM assays) or immunoassays in independent cohorts. Both the T-ALL and AML studies discussed above underscore this point: initial discovery was followed by small-scale validation, but many candidates remain to be tested in larger populations.

Compounding the challenge, there is currently a lack of harmonized protocols across laboratories for proteomic analysis. Different research groups often employ various sample preparation methods (e.g., varying depletion of abundant proteins, different digestion protocols), other mass spectrometry platforms, and distinct data analysis pipelines. These methodological variances can lead to inconsistent results; a protein biomarker discovered in one lab may not be detectable in another lab's workflow, or the measured fold-changes may differ. This lack of standardization directly affects reproducibility and thus translational validity. For a proteomic biomarker panel to be clinically useful, it must yield consistent results regardless of who performs the test or where it is performed. Achieving this consistency has proven difficult in proteomics, in contrast to, say, genetic testing, where standardized kits and protocols are more common. Recognizing this issue, the proteomics community has begun efforts to standardize and harmonize workflows. Inter-laboratory studies and quality control programs are being developed to ensure that results are reproducible across different settings [18]. For instance, a recent pan-Canadian initiative is establishing a standardized LC-MS/MS proteomics pipeline and testing its reproducibility, repeatability, and scalability across multiple cancer research centers [18]. Such efforts include developing reference materials, common calibration standards, and consensus protocols for sample processing and data analysis. By implementing uniform methodologies and rigorous quality control, researchers aim to minimize technical variability, ensuring that a given biomarker yields comparable readouts across different laboratories and instruments. Until robust reproducibility is demonstrated, it will be challenging to convince regulators and clinicians to trust proteomic biomarkers. Therefore, addressing the inter-laboratory variability problem is a critical step on the path to clinical translation. The good news is that technological improvements (e.g., more stable mass spectrometry instruments and better statistical normalization methods) and community-driven initiatives are steadily working to overcome these limitations [18,24].

## 9 Clinical translation and regulatory outlook

From a clinical perspective, demonstrating the clinical relevance and utility of LC-MS/MS-based tests will be crucial for their widespread adoption. Any proposed biomarker or panel must offer clear advantages over existing standards – for example, diagnosing leukemia at an earlier stage than current methods, or providing prognostic information that improves treatment decisions to justify incorporation into routine practice. The journey from an exciting biomarker discovery to a bedside diagnostic test is typically lengthy and demanding [17]. It involves several phases of evaluation: analytical validation (ensuring the test accurately and reliably measures the biomarker), clinical validation (showing the biomarker correlates with disease presence or outcomes in patient samples), and clinical utility assessment (proving that using the biomarker test benefits patients, such as by improving survival or reducing unnecessary treatments).

Currently, no proteomics-based leukemia diagnostic test has been approved for routine clinical use, and most identified protein biomarkers remain in the research or early validation phase [11,12]. To advance these biomarkers toward the clinic, large-scale studies – ideally multi-center clinical trials – are needed. These trials would evaluate how well a proteomic panel performs in real-world patient populations, and whether it adds value to existing diagnostic workflows. Encouragingly, there are early signs of movement in this direction. Researchers are beginning to integrate proteomic profiling into clinical studies; for example, proteogenomic analyses are being incorporated into some leukemia trials to identify markers associated with treatment response or prognosis [25,26]. Additionally, centralized laboratories with expertise in proteomics can be used to analyze patient samples in clinical trials, ensuring consistent assessment of protein markers across all trial participants [27]. These efforts will help accumulate the evidence base needed for regulatory approval.

Regulatory hurdles must also be anticipated. In many jurisdictions, diagnostic tests (especially those intended for widespread clinical use) require approval by regulatory agencies (such as the FDA in the United States or EMA in Europe). Regulatory bodies will scrutinize proteomic tests for their analytical performance (sensitivity, specificity, precision, etc.), their reproducibility (including inter-lab consistency), and their clinical validity. Meeting these criteria for a multiplex protein assay is a challenging task. Each analyte in a panel might need to be individually validated, and the assay as a whole must be standardized. Assay calibration is more complex for proteins than for DNA, due to the lack of PCR-like amplification and potential matrix effects in blood.

Furthermore, regulatory guidelines for multi-analyte assays often demand demonstrating that the test is robust and reliable in diverse clinical settings. To this end, developing standardized, easy-to-use assay formats for proteomic biomarkers will be important. This could involve converting an MS-based signature into a simpler format, such as a multiplex ELISA or an automated proteomic analyzer, that can be operated in clinical laboratories. The field is already exploring such solutions; for instance, researchers have suggested creating microfluidic LC-MS devices or miniaturized mass spectrometers that could be deployed in clinical laboratories [24]. Ultimately, achieving regulatory clearance will likely require collaboration between researchers, clinicians, and industry to ensure that proteomic tests are not only scientifically sound but also practical and reliable in a clinical laboratory environment.

Another critical consideration is cost-effectiveness. Mass spectrometry equipment and proteomic workflows are currently expensive and require specialized personnel. In a healthcare system, any new diagnostic must demonstrate that its benefits (earlier diagnosis, better risk stratification, etc.) justify its costs. If an LC-MS/MS-based test is significantly more costly or labor-intensive than existing tests, it may face hurdles in adoption. However, there is reason for optimism on the cost front: MS technology is continually becoming more efficient and cost-effective. High-throughput sample processing and automation can lower per-sample costs, and the multiplex nature of proteomic assays means that a single test could potentially replace several separate tests (for example, measuring a whole panel of protein markers at once rather than one by one). Indeed, experts have noted that mass spectrometry could become widely adopted in clinical diagnostics if issues with cost, personnel training, and workflow automation are addressed [28,29]. As automation improves and more clinical laboratories gain experience with MS-based tests, the cost barrier is expected to decrease. Demonstrating cost-effectiveness will likely involve health economics studies that compare outcomes and expenses for patients diagnosed with traditional methods versus those diagnosed with the help of the new proteomic test.

Finally, integration challenges must be tackled. Introducing LC-MS/MS proteomic assays into the clinic involves integrating them into existing laboratory and clinical workflows. Many hospital labs currently lack mass spectrometers or the trained staff to run them. This could be mitigated by centralizing testing (sending samples to specialized labs) or by introducing user-friendly MS instruments with simplified operation. Training programs for clinical laboratory scientists in proteomics will be crucial to ensure the workforce is adequately prepared. Moreover, clinicians will need education on how to interpret and use proteomic biomarker results. Integration with electronic medical record systems and decision-support tools will ensure that complex proteomic data can be understood in a clear, actionable way by healthcare providers. In the long run, multi-omics integration may provide the most value, rather than proteomic tests operating in isolation; we envision combining protein biomarkers with genomic, transcriptomic, and epigenetic markers in a single liquid biopsy platform [30,31]. Such an integrated approach could leverage the strengths of each modality, providing a more comprehensive and robust diagnostic readout (for example, DNA mutations for specificity, protein levels for functional impact, and methylation patterns for early detection). This aligns with the broader trend in oncology toward personalized medicine and may accelerate clinical acceptance. If a liquid biopsy test can offer a one-stop, comprehensive assessment (including genetic, proteomic, and other markers) with transparent clinical reporting, it becomes a beautiful tool.

In summary, translating LC-MS/MS proteomics into clinical leukemia diagnostics will require rigorous validation, standardization, and demonstration of real-world utility. The challenges are significant, but ongoing technological and methodological advances are addressing many of them. As the field progresses, we expect to see larger validation studies (including prospective clinical trials) that will clarify the actual performance of proteomic biomarkers. Concurrently, dialogue with regulators and investment in practical assay development will pave the way for eventual regulatory approvals. If successful, these efforts could lead to new diagnostic tools that detect leukemia sooner, stratify patients by risk more precisely, and ultimately guide therapy decisions, fulfilling the promise of proteomics to improve patient outcomes.

**Comparison of Liquid Biopsy Modalities:** To put proteomics into context, Table 2 provides an overview of the primary liquid biopsy approaches in leukemia, including circulating tumor DNA (ctDNA), fusion gene transcripts, epigenetic markers, and proteomic assays. Each modality offers distinct biomarkers and methods, with unique advantages and limitations in terms of detection and clinical application.

**Table 2:** Summary of major liquid biopsy technologies in leukemia and their characteristics.

Biological Marker(s)	Common Detection Methods	Key Advantages	Limitations	Current Clinical Status
Cell-free DNA fragments containing leukemia-specific mutations or gene fusions in plasma	Quantitative PCR, digital PCR, or next-generation sequencing (NGS) of plasma DNA	Non-invasive sampling; directly detects genetic alterations; sensitive monitoring of mutation burden; well-established assays for certain leukemias (e.g., BCR-ABL1 fusion).	Requires known genetic targets (cannot discover novel biomarkers); low abundance of ctDNA in some patients; detection can be confounded by age-related clonal hematopoiesis; may miss protein and epigenetic changes	Used in research and some clinical monitoring (e.g., standard of care for **BCR-ABL1** transcript monitoring in CML). Broader use in AML/ALL MRD is emerging but not yet routine.
Circulating leukemia-derived mRNA transcripts (e.g., **BCR-ABL1**, **TEL-AML1**, etc.) released into blood plasma	Reverse-transcription quantitative PCR (RT-qPCR) or targeted RNA sequencing on blood or plasma RNA	Extremely high specificity and sensitivity for known gene fusions; can detect one leukemic cell in a million via PCR; already utilized for disease monitoring (e.g., BCR-ABL1 transcript levels in CML)	Only applicable if a known fusion or transcript is present; not all leukemias have common fusion genes; RNA is less stable than DNA in blood (risk of degradation); requires careful handling of samples	Standard of care for certain leukemias (e.g., **BCR-ABL1** RT-qPCR in CML and ALL). Primarily used for MRD monitoring in known fusion-positive leukemias; being explored for other subtypes.
Cancer-associated DNA methylation signatures in cell-free DNA (often in gene promoters or other regulatory regions)	Bisulfite conversion of plasma DNA followed by methylation-specific PCR or targeted bisulfite sequencing; emerging high-throughput methods for cfDNA methylome profiling	May enable detection of leukemia even when no coding mutations are present (epigenetic alterations often occur early in leukemogenesis); provides insights into gene regulation; can complement mutation analysis.	Interpretation can be complex (background epigenetic noise, age- or clonal hematopoiesis-related changes); requires high-depth sequencing or sensitive assays due to low signal; methylation patterns may differ between leukemia subtypes; not yet standardized for routine use	Early-stage research and clinical trials. Some assays in development show that cfDNA methylation patterns can distinguish AML patients from healthy individuals. No methylation-based leukemia diagnostic is in routine clinical practice yet.
Leukemia-associated proteins and peptides in blood (e.g., secreted, shed, or leakage proteins from leukemic cells, altered host response proteins)	**Mass spectrometry** (LC-MS/MS) discovery workflows; targeted MS methods (e.g., MRM assays); multiplex immunoassays or aptamer-based assays for validation	Reflects functional and phenotypic changes of leukemia (beyond genome and transcriptome); unbiased **discovery** of novel biomarkers without prior assumptions; allows multiplex panels of proteins for increased diagnostic accuracy; potentially integrates with other “omics” markers for a comprehensive profile.	Complex analytical process (blood plasma has a high dynamic range of protein concentrations requiring special processing); results can vary with different instruments or protocols (reproducibility challenges); requires expensive equipment and specialized expertise; fewer standardized assays exist for proteins than for DNA/RNA	Currently in discovery and validation phases. Recent studies identified promising protein signatures for T-ALL and AML, but **no proteomics-based leukemia biomarker panel is yet approved for routine clinical use**. Ongoing multi-center studies aim to validate proteomic panels, and proteomics is being integrated into research trials to evaluate its clinical utility.

## 10 Conclusion

Liquid biopsy-based leukemia diagnosis is a rapidly advancing field that has the potential to transform the way we detect and manage these cancers. LC-MS/MS has proven to be a linchpin technology for unbiased protein biomarker discovery in liquid biopsies. By identifying proteomic signatures in the blood that correlate with early leukemia, this approach offers a path toward less invasive and earlier diagnosis of diseases like T-ALL and AML. Studies conducted between 2022 and 2025 have demonstrated that blood-based proteomic profiles can distinguish between leukemia patients and healthy individuals with high accuracy and have even pinpointed small panels of proteins with potential clinical utility [32,33]. The advantages of LC-MS/MS – including its sensitivity, multiplexing, and specificity position it as a strong complement to traditional genomic tools and bone marrow examinations in leukemia diagnostics. However, realizing the full clinical impact of LC-MS/MS will require surmounting validation and implementation challenges. As the field advances, we can expect to see larger validation studies and the integration of proteomic biomarkers with other molecular data to enhance diagnostic accuracy. Encouragingly, the trend in oncology is toward personalized medicine, and liquid biopsy approaches fit naturally into this paradigm by enabling frequent, patient-friendly monitoring and tailored treatment decisions [34]. An integrated multi-omic liquid biopsy would allow patients to receive earlier disease diagnosis, refined risk stratification, and subsequent targeted treatments with unprecedented precision [35]. In summary, LC-MS/MS-based proteomics opens new horizons for leukemia diagnosis and prognosis, offering hope for earlier and less invasive detection, ultimately improving patient outcomes.

## Ethics approval statement

Ethics approval was not required for this review.

## Ethical approval/patient consent

Not Applicable.

## Declaration of competing interest

The authors declare the following financial interests/personal relationships, which may be considered as potential competing interests.
